# Accuracy of mRNA HPV Tests for Triage of Precursor Lesions and Cervical Cancer: A Systematic Review and Meta-Analysis

**DOI:** 10.1155/2019/6935030

**Published:** 2019-06-11

**Authors:** Ana Cristina L. Macedo, João Carlos N. Gonçalves, Daniela Vicente Bavaresco, Antonio José Grande, Napoleão Chiaramonte Silva, Maria Inês Rosa

**Affiliations:** ^1^Translational Biomedicine Laboratory, Graduate Program in Health Sciences, University of Southern Santa Catarina (UNESC), Criciúma, SC, Brazil; ^2^Laboratory of Evidence in Health, Medicine and Health Sciences, University of State of Mato Grosso do Sul, Campo Grande, MS, Brazil

## Abstract

**Objective:**

This systematic review evaluates the accuracy of the mRNA HPV biomarker in cervical smears to identify cervical intraepithelial neoplasia (CIN) 2 or 3 and cervical cancer.

**Data Source:**

Eligible studies were identified by performing a search of electronic databases on Medline via Pubmed, Lilacs, Cochrane Library, Embase, and Grey literature for papers published between January 1990 and June 2018.

**Study Eligibility Criteria:**

As no randomized studies were identified, this review focuses on observational studies in which the mRNA HPV diagnostic test was compared to a histopathology reference standard. We analyzed studies that included women screened for cervical cancer using mRNA HPV.

**Study Appraisal and Synthesis Methods:**

After screening, 61 studies including 29,674 patients met the inclusion criteria and were analyzed. Dichotomization was performed by defining CIN2 or worse (CIN2+) versus CIN1, HPV infection, and normal (CIN 1-). The analysis was discriminated by the following tests: Aptima, PreTect HPV Profeer, NucliSens EasyQ HPV, OncoTect, and Quantivirus.

**Results:**

Analyzing by technique, Aptima, with 28 studies, exhibited superior performance, showing for the outcomes CIN2+ and CIN3+ an AUC of 0.88 (0.82-0.95) and 0.91 (0.84-0.99), a pooled sensitivity of 92.8% (95%CI 91.9-93.7) and 95.6% (95%CI 94.5-96.5), and a pooled specificity of 60.5% (95%CI 59.8-61.3) and 61.9% (95%CI 61.1-62.7), respectively.

**Conclusion:**

This study supports the current hypothesis that the mRNA HPV assay is an adequate tool for secondary cervical cancer screening.

## 1. Introduction

Cervical cancer is the third most common malignancy in women and fourth in mortality worldwide. In 2012, there were 406,210 diagnosed cases and 265,672 deaths [1]. In the United States, there were 12,578 new cases and 4,115 deaths in 2014 [2]. Of note, screening tests for cervical cancer make this disease one of the most easily preventable malignant tumors. Worldwide, cervical cancer screening is accomplished using the Papanicolaou test, which looks for cytological abnormalities. If identified, the patient will be referred for colposcopy and targeted biopsies. Given consensus regarding the causal role of high-risk human papillomavirus (HR HPV) in the development of cervical cancer [3], DNA hrHPV assays have been incorporated as a screening method in some developed countries [4–6]. HPV is the number one most common infectious agent related to cancer development in women, and it is estimated that 570,000 cases of cancer arose from this infection in 2012, including anogenital and oropharynx cancers. Currently, the following HPV strains are considered high risk with respect to cervical cancer development: 16, 18, 31, 33, 35, 39, 45, 51, 52, 56, 58, and 59 [1, 7].

Screening strategies should balance potential benefits and potential harm from intervention. DNA hrHPV tests exhibit high sensitivity with low specificity when the outcome is a precancerous lesion [4, 6]. Maintaining a 3-year interval between screening visits is a good safety measure, but it increases unnecessary routing to colposcopy with a potential rise in cost and overtreatment [4, 6]. As a result, some countries are adopting a 5-year interval [4, 6]. In this scenario, an assay with good accuracy and improved specificity should be associated with or used alone in primary screening. Previous studies reported that mRNA HPV tests, which reveal current HPV oncogene expression and evidence of its deregulation per detection of viral proteins, possess these characteristics [66, 67].

The present systematic review assesses the accuracy of mRNA HPV tests globally that have been submitted to sensitivity analysis and, when available, compared with the DNA hrHPV test and cytology. The prespecified hypothesis is that mRNA HPV exhibits acceptable accuracy and high specificity for detection of high-grade squamous intraepithelial lesion (HSIL) or cervical intraepithelial neoplasia (CIN) 2 or 3, precancerous lesions, and cervical cancer.

## 2. Methods

We performed a systematic review according to a prospective protocol using PRISMA statement guidelines. This review protocol is registered at PROSPERO (International prospective register of systemic reviews, http://www.crd.york.ac.uk/prospero; CRD 2015: CRD42015020232).

### 2.1. Identification of Studies

Eligible studies were identified by performing a search of electronic databases on Medline via Pubmed, Lilacs, Cochrane Library, Embase, and Grey for papers published from January 1990 to October 2017. A search on clinical trials was not performed because this database includes intervention trials and is used primarily for intervention systematic reviews and not for diagnostic reviews. The medical subject headings (MeSH) and text words for the terms: “cervical cancer”, “cervical dysplasia”, “squamous intraepithelial lesion”, “cervical intraepithelial neoplasia”, “CIN”, “screening” and “RNAm HPV” were entered. No language restrictions applied. Reference lists of all available primary studies were reviewed to identify additional relevant citations.

### 2.2. Study Selection

As no randomized studies were identified, this review focused on observational studies in which the mRNA HPV diagnostic test was compared to a histopathological reference standard. All included studies were cross-sectional or, if cohort study, it was included only if biomarkers, cytology, and histopathology have been available in baseline, to characterize a cross-sectional data.

### 2.3. Patients

We analyzed studies that included women who were screened for cervical cancer in secondary settings, that is, testing performed after someone has had an abnormal result by cytology or HPV testing. When the study was originally from primary screening, only the sample with abnormalities and that had been forwarded to colposcopy was considered. Additionally, when only considering samples submitted for colposcopy, whenever possible, only biopsied samples were included. These variables were subsequently considered in the sensitivity analysis.

### 2.4. Index Test

The index test was an mRNA HPV test from a sampling of a cervical smear. Positive and negative reads were assigned according to the cut-off points proposed by the manufacturers.

As alternative tests, the accuracy of DNA hrHPV tests was extracted when applied to the same sample used for the mRNA test.

The exclusion criteria for index tests applied in tissue fragments. Studies in which all specimens were diagnosed as cancer were excluded, since there were no false positives or true negatives.

### 2.5. Reference Standard

The reference test was histologic evaluation of tissue in paraffin-embedded sections using the same Bethesda System classification.

### 2.6. Data Extraction

This study was independently reviewed by two investigators (MIR, ACM). Disagreements with regard to study inclusion or exclusion were initially resolved by consensus. When consensus was not attained, disagreements were resolved by a third reviewer (JCG).

### 2.7. Assessment of Methodological Quality

Methodological quality assessment of studies for diagnostic accuracy was performed according to criteria from the Quality Assessment of Diagnostic Accuracy Studies (QUADAS-2). These criteria assess the quality of included studies in terms of risk of bias and concerns regarding applicability over four domains [68].

### 2.8. Statistical Analysis

A 2 x 2 contingency table was constructed for each selected study. Rates were calculated as true positive (TP), false positive (FP), true negative (TN), and false negative (FN). When any cell containing “0" was present in the contingency table, 0.5 was added to all cells in all studies to facilitate calculations. Dichotomization of the contingency tables was performed by defining two categories: (1) CIN2 or worse versus CIN 1 and normal and (2) CIN 3 or worse versus CIN1 and normal (excluding CIN2 from the analysis, since we do not believe that CIN2 can be seen as a false positive).

For all studies, we calculated the true-positive rate (TPR; sensitivity), specificity, false-positive rate (FPR; 1 – specificity), and the diagnostic odds ratio (DOR). The DOR, which relates to different combinations of sensitivity and specificity, was calculated by (sensitivity/(1-specificity))/((1-sensitivity)/specificity)) [69]. A DOR > 1 indicated the assay had discriminative power. The DOR describes the odds of the positive test results in participants with disease compared with the odds of positive test results in those without disease. Bivariate analysis was used to calculate pooled estimates of sensitivity, specificity, and DOR with 95% confidence intervals (CIs) for summary estimates [70].

To analyze the accuracy of HPV mRNA, the area under the curve (AUC) was calculated from the hierarchical summary receiver-operator curves (HSROC). AUC values ≥ 0.5, 0.75, 0.93, and 0.97 were considered to represent fair, good, very good, and excellent accuracy, respectively [71].

Heterogeneity of both sensitivity and specificity across the studies was tested using a *χ*^2^ analysis, with a *χ*^2^ p-value < 0.05 considered heterogeneous. As an alternative method to explore heterogeneity, the I^2^ index was also utilized. The I^2^ index presents the percentage of total variation across studies due to heterogeneity rather than chance; I^2^ values of 75% or greater were considered substantial heterogeneity [70].

To analyze publication bias, inverted funnel plots of the logarithmic odds ratio (OR) of individual studies were plotted against the sample size. The robustness of the results was tested by repeating the analysis with a different statistical model (random effects model). The meta-analysis was performed using Metadisc® and Review Manager® (RevMan) version 5.2 software [72, 73].

## 3. Results

### 3.1. Study Identification and Eligibility

**Figure 1:**
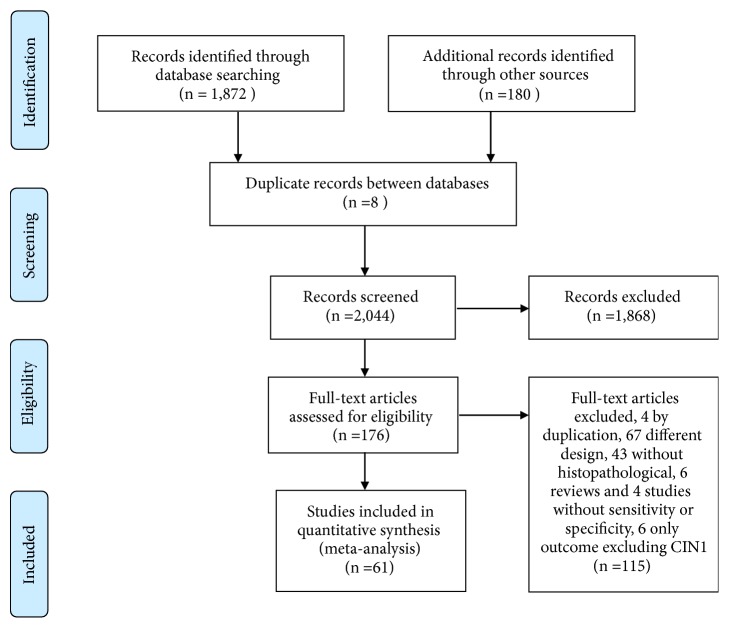
PRISMA Flowchart of the search strategy.

Among the 2,052 studies identified from electronic database searches and reference lists, we excluded 1,868 published studies through title and abstract screening (Figure 1). One hundred seventy-six full-text studies were then retrieved. Of those, 107 studies were excluded after further scrutiny. A complete list of excluded studies is available from the authors.

### 3.2. Study Descriptions

Sixty-one primary studies were included [8–65, 74–76] in cytology secondary analyses. Of the main analysis, 60 studies informed the major outcome, CIN1- vs. CIN2+, and 39 studies have shown CIN1- vs. CIN3+. A total of 29,674 patients met the criteria for inclusion and were analyzed. The main characteristics of the included studies are shown in Table 1.  Table 2 shows the sum contingency tables with regard to the different techniques applied for CIN1- vs. CIN2+ and CIN1- vs. CIN3+. The contingency tables per study may be requested from the authors.

### 3.3. Quality Assessment

**Figure 2:**
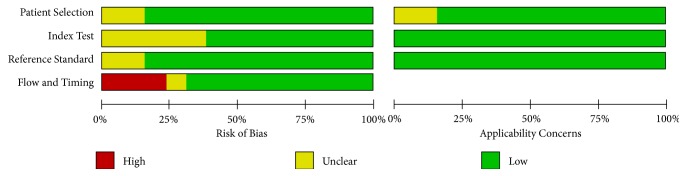
Quality Assessment of Diagnostic Accuracy Studies (QUADAS-2).

QUADAS-2 was performed considering the following categories: index and reference test, flow, and timing (Figure 2). For the index and reference test, most studies did not mention blinding of the pathologists and were classified as “unclear." In 37.7%, the verification of the histopathological examination was partial; that is, women with normal colposcopy were not biopsied, as shown in Table 1. In addition, all included studies used a histopathological test as a reference, and the index tests were clearly cited. Therefore, “concern” with these items was low. For flow and timing, six studies did not cite the interval between the index and referenced tests [12, 24, 25, 35, 37, 75], and in one, the interval was considered inadequate because it was from a cohort that did not show separate baseline and follow-up results [27]. In cohort studies, we considered the results of the baseline whenever possible.

### 3.4. Accuracy of HPV mRNA

The accuracy (sensitivity, specificity, AUC, DOR, and sum contingency tables) of HPV mRNA tests stratified by kit identified in this systematic review is discriminated in Table 2.

**Figure 3:**
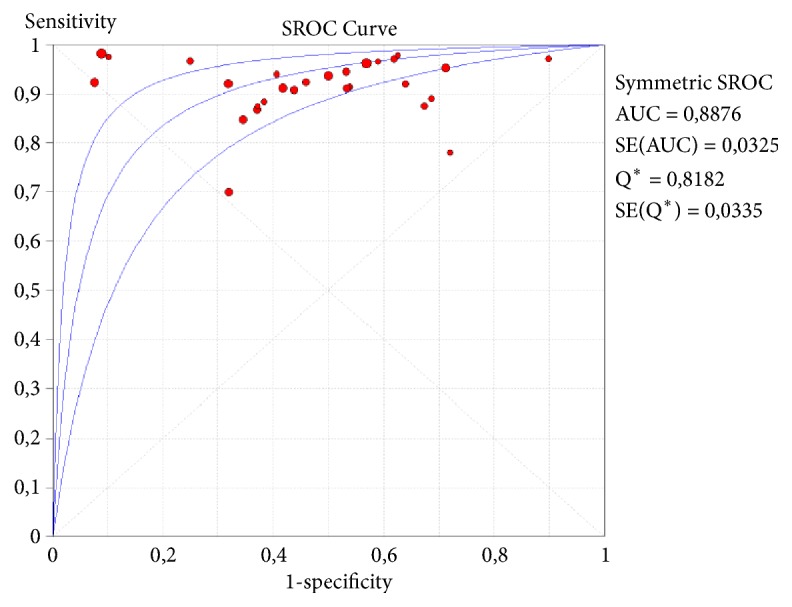
Forest plot of sensitivity and specificity of cervical cytology.

Different techniques are available, based on identification of HPV mRNA transcription, mainly of E6 and E7 oncogenes. In this systematic review, five main tests were identified. Aptima (Hologic Gen-Probe, San Diego, CA, USA) is a target amplification assay utilizing transcription-mediated amplification (TMA) for qualitative detection of viral polycistronic E6/E7 mRNA from 14 high-risk HPV types [77]. PreTect HPV-Proofer (NorChip AS, Klokkarstua, Norway) is a real-time multiplex assay that uses nucleic acid sequence-based amplification (NASBA), a sensitive transcription-based amplification system (TAS) for the specific in vitro replication of mRNA. NucliSens EasyQ HPV (bioMérieux, The Netherlands) is based on the original PreTect Proofer assay with the addition of the NucliSENS hardware platform and the software for NASBA measurements and data analysis, both identifying the same five most frequently recognized HPV types [78]. OncoTect (IncellDxTM, Inc. Menlo Park, CA, USA) combines two techniques, called in situ hybridization and flow cytometry. Finally, the Quantivirus HPV E6/E7 RNA 3.0 assay (DiaCarta, Hayward, CA, USA) detects E6/E7 mRNA of 13 high-risk and 6 low-risk types and is a sandwich nucleic acid hybridization procedure using chemiluminescent detection of mRNA molecules that are hybridized to DNA probes [65]. Aptima, with 28 studies, exhibited superior performance, with the best sensitivity, near from Hybrid Capture 2, and higher specificity, comparing to this assay, as shown ahead. Its SROC is shown in Figure 3.

We considered the importance of describing the results divided by age; however, few studies [13, 49] discriminated between the over and under 30 years of age category, and there were no important differences in this small sample (data not shown).

### 3.5. Comparing HPV mRNA to hrHPV DNA

Some studies applied two or more assays to the same sample, making it possible to compare them. In the outcome CIN1- vs. CIN2+, comparing Aptima to Hybrid Capture 2 (HC2, Qiagen, Gaithesburg, MD, USA), a DNA hrHPV test, fourteen studies were available [8, 12–14, 19, 20, 25–29, 31, 32, 35]. The pooled sensitivity identified was 93.9% (95%CI 92.8-94.8) and 94.3% (95%CI 93.3-95.2), pooled specificity of 61.5% (95%CI 60.6-62.7) and 51.3% (95%CI 50.2-52.4), the DOR was 15.96 (95%CI 10.14-25.17) and 12.55 (95%CI 92.33-17.07), and the AUC was 0.90 (0.80-1) and 0.91 (0.88-0.95), respectively, for Aptima and Hybrid Capture 2 (Table 3).

### 3.6. Sensitivity Analysis

Discerning by complete verification of the reference test or partial verification, we identified that all samples were biopsied in 38 studies, whereas in 23 studies, they were not (Table 1). In the completely biopsied sample group, the pooled sensitivity was 86.9% (95%CI 85.4-88.2) and the pooled specificity 64.8% (95%CI 63.7-65.8). The DOR was 10.49 (95%CI 6.94-15.85), and the AUC was 0.85 (95%CI 0.79-0.92). In contrast, in the partially biopsied sample group in which women with normal colposcopy were not biopsied, the pooled sensitivity was 80.2% (95%CI 78.8-81.5) and pooled specificity 72.6% (95%CI 71.7-73.5). The DOR was 13.96 (95%CI 9.798-19.91), and the AUC was 0.86 (95%CI 0.82-0.90). This difference is potentially caused by the higher frequency of Aptima studies in the “all biopsied” group, 55.2% vs. 30.4%, as in comparison, this assay has superior sensitivity, as shown above.

## 4. Discussion

The aim of this systematic review was to evaluate the accuracy of the biomarker HPV mRNA as a means to identify CIN and cervical cancer, a disease with a high prevalence, primarily in low-resource countries. In this analysis, we show 60 studies with the same outcome, making this the most extensive review on the topic to our knowledge.

Two systematic reviews have already been performed analyzing the HPV mRNA test accuracy. Burger et al., in 2011, conducted a systematic review predominately including studies from nonspecific secondary screening [67], and Verdoodt et al., in 2013, included studies with minor abnormal cervical cytology [66]. The first one included 11 studies and concluded that sensitivities ranged from 41.0% to 86.0% and from 90.0% to 95.0% for the PreTect Proofer/NucliSENS Easy Q and Aptima assay, respectively. Specificities ranged from 63.0% to 97.0% and from 42.0% to 61.0% for the same assays, respectively. In our study, the greater number of primary studies led to a wider range of results but maintained the same trend. In a study by Verdoodt et al., which included 10 studies using PreTect Proofer/NucliSENS Easy Q, they concluded that the pooled sensitivity was 75.4% and 76.2% and the pooled specificity was 77.9% and 74.2%, for the triage of ASC-US and LSIL, respectively. These are very close to our results, except that, in our sample, NucliSens EasyQ HPV exhibited a lower specificity.

One of the most promising algorithms is in effect primary screening with the hrHPV DNA test, which has superior sensitivity, and use of the HPV mRNA test, due to its high specificity and the possibility to perform the test with the same sample without the need for patient return. Another possibility is to substitute hrHPV DNA and cytology for HPV mRNA testing. Zappacosta et al., 2015, published a prospective study that compared the cost and effectiveness of three strategies for management of ASC-US and LSIL cytology patients: immediate colposcopy, triage with the hrHPV DNA test, and the HPV mRNA test [79]. They concluded that the HPV mRNA test exhibited overall percentage agreement with histological diagnosis of 89.8%, and as to the AUC, the hrHPV DNA test was 0.79 and the HPV mRNA test 0.92. Cotesting with HPV DNA and mRNA, in comparison with immediate referral, reduced colposcopy referral by 77.5% and by 54.5% in comparison with hrHPV DNA alone. An American study comparing cotesting cytology and hrHPV DNA (n=1,856) or HPV mRNA (n=1,651) in ASC-US cytology samples concluded that the change in the hrHPV detection methodology from HC2 to Aptima has led to a 21% reduction in colposcopy referrals and is more cost-effective for patient care [80]. A multicenter trial with 5,006 women undergoing routine screening in France comparing an HPV mRNA test (Aptima), an hrHPV DNA test (HC2), PCR genotyping, and cytology (LBC) already illustrated that Aptima exhibits the highest absolute risk of both histological endpoints and detected 5% to 15% more CIN3+ and CIN2+ lesions, respectively, than did cytology. Compared with the HC2 assay, the relative risk of Aptima was 24% to 29% higher, with a significant difference in CIN2+ detection, concluding that Aptima is a suitable option for primary cervical cancer screening [81]. In our study, the accuracy was greater for Aptima, when compared to hrHPV DNA tests, suggesting that this could be an adequate substitute, especially considering improvements in specificity. In secondary screening, a test with improved specificity would be more useful, like OncoTect or PreTect HPV Profeer.

Great heterogeneity in sensitivity and specificity was found among studies. This could be explained by different samples and different frequencies of CIN in each population. We performed sensitivity analysis using different screening criteria and studies with partial or complete verification of the reference test, to try and detect confounding factors, but the results retained high heterogeneity (data not shown).

In conclusion, this study supports the current hypothesis that HPV mRNA assays are an adequate tool in the secondary screening of cervical cancer.

## Figures and Tables

**Table 1:** Characteristics of included studies.

AUTHORS MAIN ANALYSIS	YEAR	COUNTRY	INCLUSION CRITERION	AGE MEAN (RANGE)	N TOTAL	N BENIGN	N CIN2+	N CIN3+	DNA HRHPV TEST (IF PRESENT)	MRNA HPV TEST	VERIFICATION BY HISTOPATHOLOGY*∗*
ALAGHEHBANDAN ET AL.	2013	Canada	abnormal cytology	30.7	1289	929	360	NI	HC2	PreTect HPV-Proofer	Partial
ANDERSSON ET AL.	2006	Sweden	abnormal cytology	35.3 (23-60)	71	32	39	22	RT-PCR	NucliSens EasyQ HPV	complete
BENEVOLO ET AL.	2011A	Italy	HSIL in cytology	39.5 (18-83)	139	105	34	NI	HC2 or PCR (HPV MX BIO)	PreTect HPV-Proofer	Partial
BINNICKER ET AL.	2014	USA	abnormal cytology	NI	370	289	81	41	HC2	Aptima	complete
CASTLE ET AL.	2007	USA	ASC-US in cytology	NI	531	426	105	54	HC2	Aptima	complete
CASTLE ET AL.	2015	US and England	ASC-US in cytology	34.2 (21-28)	713	634	79	33	none	Aptima	complete
CATTANI ET AL.	2009	Italy	not specified	35 (20-77)	143	84	59	41	HC2	NucliSens EasyQ HPV	complete
CHERNESKY ET AL.	2017	Canada and USA	abnormal cytology or DNA+	36.1 (21-80)	1350	1203	147	71	COBAS4800	Aptima	complete
CLAD ET AL.	2011	Germany	abnormal cytology	NI	424	172	252	163	HC2	Aptima	Partial
COQUILLARD ET AL.	2011	USA and Spain	not specific	NI	217	187	73	30	HC2	OncoTect	complete
CUSCHIERI ET AL.	2013	UK	abnormal cytology	29.3 (25-38)	1366	987	379	175	HC2	Aptima	Partial
CUZICK ET AL.	2013	UK	abnormal cytology	37 (20-66)	119	79	40	19	HC2	Aptima/PreTect HPV Proofer	complete
DOCKTER ET AL.	2009	USA	not specified	NI	753	612	141	87	HC2	Aptima	Partial
DUVLIS ET AL.	2015	Republic of Macedonia	not specific	(19-78)	61	32	29	NI	PCR	NucliSens EasyQ HPV	Partial
EVANS ET AL.	2014	USA	Any CIN in histopathological	28.8 (17-57)	86	32	54	32	CISH	RNA scope 2.0 (CISH)	Partial
GALAROWICZ ET AL.	2012	Poland	not specified	37,8 (19-81)	85	49	36	NI	HC2	NucliSens EasyQ HPV	Partial
GE ET AL.	2017	USA	not specified	NI	175	146	29	NI	none	Aptima	complete
GE ET AL.	2018	USA	not specified	NI	603	500	103	NI	none	Aptima	complete
GUO ET AL.	2014	China	ASC-US/ LSIL	34 (21-69)	411	339	72	17	HC2	Aptima	complete
HALFON ET AL.	2010	France	abnormal cytology	38 18-77	112	75	37	NI	HC2	NucliSens EasyQ HPV	Partial
HOVLAND ET AL.	2010	Norway, Belgium, Sweden, Congo, Netherlands	not specific	37 (25-60)	313	297	16	NI	PCR	PreTect HPV-Proofer	complete
IFTNER ET AL.	2015	Germany	abnormal cytology, mRNA+ or DNA+	(30-60)	603	513	90	43	HC2	Aptima	complete
JOHANSSON ET AL.	2015	Sweden	ASC-US/ LSIL	42 (35-68)	342	236	106	43	none	Aptima	complete
KOILOPOULOS ET AL	2012	Greece	ASC-US/ LSIL	38	79	37	42	12	none	NucliSens EasyQ HPV and OncoTect	Partial
KOTTARDI ET AL.	2011	Greece	abnormal cytology	(21-65)	189	146	43	16	PCR (CLART2)	OncoTect	Partial
LI ET AL.	2017	China	ASC-US in cytology	NI	189	121	68	33	HC2	Quantivirus	complete
LIE ET AL.	2005	Norway	abnormal cytology	35 (19-85)	383	92	291	NI	HC2	PreTect HPV-Proofer	complete
LIU ET AL.	2014	China	abnormal cytology or DNA+	NI	92	35	57	56	Quantivirus	Quantivirus	Partial
LIU ET AL.	2017	China	ASC-US in cytology	>30	312	159	153	79	none	Quantivirus	complete
MOLDEN ET AL.	2005	Norway	HSIL in cytology	48.9 (30-91)	23	9	14	NI	none	PreTect HPV Proofer	complete
MONSONEGO ET AL.	2011	France	abnormal cytology, mRNA+ or DNA+	(20-65)	1113	1012	101	27	HC2	Aptima	complete
MUANGTO ET AL.	2016	Thailand	abnormal cytology	96.4%>30 years	1362	1349	13	12	Cervista	Aptima	Partial
OLIVEIRA ET AL.	2013	Portugal	not specified	34.6 (18-73)	554	259	295	NI	HC2	NucliSens EasyQ HPV	Partial
OVESTAD ET AL.	2011	Norway, USA, China Netherlands	ASC-US/ LSIL	40 (25-69)	121	76	45	NI	COBAS4800	PreTect HPV-Proofer/ Aptima	complete
PADALKO ET AL.	2013	Belgium	ASC-US in cytology	NI	35	8	27	NI	PCR	NucliSens EasyQ HPV	complete
PEREZ CASTRO ET AL.	2013	Spain	HSIL in cytology	36.9 (20-71)	49	44	5	NI	none	NucliSENSEasyQ	Partial
PERSSON ET AL.	2014	Sweden	ASC-US/ LSIL	32.8	205	132	73	36	Linear Array	Aptima	complete
PIERRY ET AL.	2012	USA	abnormal cytology	46%>30	246	201	45	15	none	OncoTect	Partial
RATNAM ET AL.	2009	Canada	abnormal cytology	NI	831	591	240	NI	HC2	PreTect HPV-Proofer/ Aptima	complete
RATNAM ET AL.	2010	Canada	abnormal cytology	31 (15-80)	1551	1149	402	NI	HC2	PreTect HPV-Proofer	Partial
RATNAM ET AL.	2011	Canada	abnormal cytology	36.3 (16-81)	1418	1017	401	281	HC2	Aptima	Partial
REBOJI ET AL.	2014	Denmark	abnormal cytology	NI	259	140	119	84	HC2	Aptima	complete
REID ET AL.	2015	USA and UK	not specific	44.2 (30-89)	818	798	20	11	HC2	Aptima	Partial
REN ET AL.	2017	China	ASC-US in cytology	38.5 (19-68)	160	129	31	NI	HC2	Quantivirus	complete
REUSCHENBACH ET AL.	2010	Germany	abnormal cytology	36 (28-44)	237	73	164	110	HC2	Aptima	complete
SHEN ET AL.	2013	China	not specified	37 (16-77)	75	58	17	NI	HC2	Quantivirus	complete
SORBYE ET AL.	2011	Norway	LSIL in cytology	NI		297	228	69	none	PreTect HPV-Proofer	complete
STATHOPOULOU ET AL.	2014	Greece	not specified		1039	591	53	24	none	NASBA/ OncoTect	Partial
STOLER ET AL.	2013	US and England	ASC-US in cytology	31 (21-71)	740	649	91	41	HC2	Aptima	complete
SZAREWSKI ET AL.	2012	UK, USA and France	abnormal cytology	29 (26-35)	911	552	359	224	HC2	PreTect HPV-Proofer/ Aptima	complete
TROPÉ ET AL.	2009	Norway	HSIL+ in cytology	37 (17-76)	1379	736	643	508	Amplicor	PreTect HPV-Proofer	Partial
TROPÉ ET AL.	2012	Norway	ASC-US/ LSIL	39.6 (18-83)	665	565	100	60	Amplicor	PreTect HPV-Proofer	Partial
TUNEY ET AL.	2017	Turkey	abnormal cytology	42.4 (22-89)	25	15	NI	10	PCR	NucliSens EasyQ HPV	complete
VALASOULIS ET AL.	2014	UK	HSIL+ in cytology	37.8 (21-63)	189	100	89	NI	PCR (CLART2)	NASBA/ OncoTect	complete
VALENÇA ET AL	2015	Brazil	HSIL+ in cytology	35.3	111	39	72	NI	none	NucliSENSEasyQ	complete
VIRTANEN ET AL.	2016	Finland	abnormal cytology	(18-86)	330	263	67	NI	HC2	Aptima	complete
WALDSTROM ET AL.	2011	Denmark	ASC-US in cytology	42.2 (30-69)	169	121	48	27	Linear Array	Aptima	complete
WALDSTROM ET AL.	2013	Denmark	LSIL in cytology	32.3 (16-65)	469	382	87	46	none	Aptima	complete
WESTRE ET AL.	2016	Norway	ASC-US/ LSIL	39	162	126	36	NI	COBAS	PreTect HPV-Proofer	Partial
WOJCIECH ET AL.	2012	Poland	abnormal cytology, mRNA+ or DNA+	45 (25-65)	421	339	82	NI	COBAS4800	NucliSens EasyQ HPV	complete
WU ET AL.	2010	China and USA	abnormal cytology or DNA+	35 (25-59)	2000	1973	27	15	HC2	Aptima	complete

CIN: cervical intraepithelial neoplasia.

If the information was available, N total and N benign included CIN1. NI: not informed. *∗*Verification by histopathology: studies with partial verification only performed biopsy in women with colposcopy lesion.

**Table 2:** Accuracy of mRNA HPV for detection of Cervical Intraepithelial Neoplasia (CIN) in histopathological, Pooled and discerning by mRNA HPV test. Outcomes: CIN1- vs. CIN2+ and CIN1- vs. CIN3+.

*Test*	*All mRNA HPV assays pooled*	*Aptima* % (IC 95%)	*NucliSens EasyQ HPV* % (IC 95%)	*OncoTect* % (IC 95%)	*PreTect HPV Proofer* % (IC 95%)	*Quantivirus* % (IC 95%)
*CIN1- vs. CIN2+*						
Sensitivity	83.3 (82.9-84.6)	92.8 (91.9-93.7)	75.9 (72.7-78.9)	72.4 (67.5-76.9)	73.2 (71.5-74.9)	86.6 (82.4-90.1)
Specificity	65.2 (64.5-65.8)	60.5 (59.8-61.3)	61.5 (58.5-64.5)	79.5 (77.4-81.5)	79.4 (78.3-80.5)	38.9 (35.1-42.8)
DOR	10.54 (8.35-13.29)	12.53 (8.97-17.52)	5.48 (3.37-8.89)	13.83 (6.40-29.86)	13.21 (8.55-20.41)	4.71 (2.59-8.57)
AUC	0.84 (0.81-0.87)	0.88 (0.82-0.95)	0.76 (0.69-0.82)	0.87 (0.82-0.92)	0.84 (0.79-0.89)	0.80 (0.66-0.95)
TP	5,840	3,220	578	267	1,992	278
FP	7,910	6,177	392	319	1,125	390
FN	1,131	248	184	102	728	43
TN	14,793	9,470	627	1,238	4,337	248
*N total*	*29,674*	*19,115*	*1,781*	*1,926*	*8,182*	*959*

*CIN1- vs. CIN3+∗*						
Sensitivity	86.1 (84.8-87.3)	95.6 (94.5-96.5)	83.5 (73.9-90.7)	85.2 (77.4-91.1)	67.6 (64.3-70.7)	85.1 (78.8-90.1)
Specificity	65.5 (64.8-66.2)	61.9 (61.1-62.7)	64.1 (55.3-72.3)	78.6 (77.6-80.6)	83.9 (82.2-85.5)	41.5 (36.9-46.2)
DOR	18.93 (12.44-28.82)	21.45 (12.40-37.11)	9.67 (0.931-100.54)	23.33 (8.07-67.49)	19.57 (4.36-87.85)	7.28 (4.11-12.88)
AUC	0.88 (0.84-0.92)	0.91 (0.84-0.99)	0.78 (0.56-0.99)	0.84 (0.78-0.89)	0.71 (0.67-0.76)	0.79 (0.68-0.89)
TP	2,494	15	71	98	579	143
FP	6,238	174	98	351	311	261
FN	403	0	14	17	278	25
TN	11,854	1,799	297	1,292	1,621	185
*N total*	*20,989*	*1,988*	*480*	*1,758*	*2,789*	*614*

*∗*Excluding CIN2 from analysis. CIN: cervical intraepithelial neoplasia; CI: Confidence interval; DOR: diagnostic odds ratio; AUC: area under the curve; TP: true positive; FP: false positive; FN: false negative; TN: true negative.

Included studies CIN1- vs. CIN2+: Aptima [8–35]; NucliSens EasyQ HPV [36–46]; OncoTect [41, 47–51]; PreTect HPV Proofer [14, 23, 25, 31, 52–61]; Quantivirus [45, 62–65].

CIN1- vs. CIN3+: Aptima [8–15, 18–22, 24, 26–31, 33–35]; NucliSens EasyQ HPV [36, 37, 41, 46]; OncoTect [41, 47–50]; PreTect HPV Proofer [14, 31, 59–61]; Quantivirus [62–64].

**Table 3:** Accuracy of Aptima for detection of Cervical Intraepithelial Neoplasia (CIN) in histopathological, compared to a DNA hrHPV test (Hybrid Capture 2), in the same sample. Outcome: CIN1- vs. CIN2+.

	*Aptima* % (IC 95%)	*Hybrid Capture 2* % (IC 95%)
Sensitivity	93.9 (92.8-94.8)	94.3 (93.3-95.2)
Specificity	61.7 (60.6-62.7)	51.3 (50.2-52.4)
DOR	15.96 (10.14-25.17)	12.55 (92.33-17.07)
AUC	0.90 (0.80-1)	0.91 (0.88-0.95)
TP	2,184	2,206
FP	3,243	4,092
FN	143	133
TN	5,216	4,312
*N total*	*10,786*	*10,743*

CIN: cervical intraepithelial neoplasia; CI: Confidence interval; DOR: diagnostic odds ratio; AUC: area under the curve; TP: true positive; FP: false positive; FN: false negative; TN: true negative.

*∗*Small differences between Aptima e HC2 total is due to losses in three studies in HC2 sample: Clad et al., 2011, Monsonego et al., 2011 and Reid et al., 2015.

## Additional Points

*Recommendations*. Although this systematic review clearly shows the accuracy of HPV mRNA for cervical cancer screening, additional prospective and randomized studies are necessary in order to establish cost-effectiveness and possible changes in screening guidelines.
